# Supplementary material for: Identification of photoperiod-induced specific miRNAs in the adrenal glands of Sunite sheep (Ovis aries)
Source: Front Vet Sci. 2022 Jul 22;9:888207. doi: 10.3389/fvets.2022.888207 (PMC9354845; doi:10.3389/fvets.2022.888207)
Supplement: Supplementary file 1 [file Data_Sheet_1.ZIP › Supplementary file/miRDeep2 file.zip!/miRDeep2 file/Novel_Structure/SPLP42A2/Novel_363.pdf]

Provisional ID	:	NC_019459.2_5423
Score total	:	2.4
Score for star read(s)	:	-1.3
Score for read counts	:	0
Score for mfe	:	2.1
Score for randfold	:	1.6
Score for cons. seed	:	
Total read count	:	14
Mature read count	:	12
Loop read count	:	0
Star read count	:	2

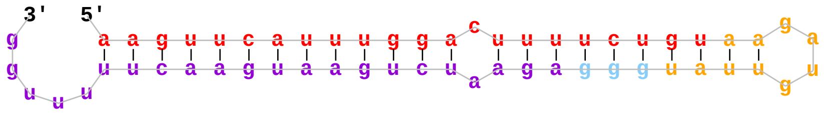

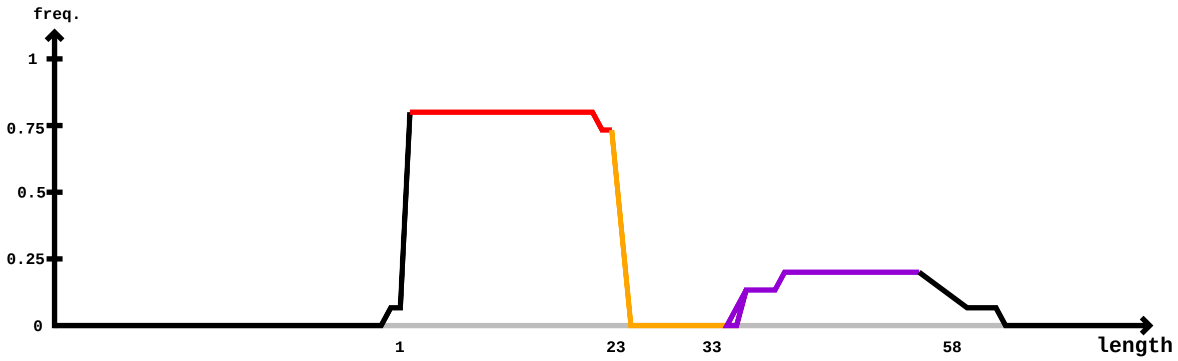

Mature Star

```
5'- aguuuguuuauucuauauauauuggauuggucaaaaaguucauuuggacuuuucuguaagauguuauggggagaaucugaaugaacuuuuuggccaaucccaauaagcaguuu   -3'    obs
    aguuuguuuauucuauauauauuggauuggucaaaaaguucauuuggacuuuucuguaagauguuaugggagaaucugaaugaacuuuuuggccaaucccaauaagcaguuu          exp
    ...(((((((((...........(((((((((((((((((((((((((.((((((((((....)))))))))).))))))))))))))))))))))))).)))))))))...   reads      mm          sample
    .................................aaaaguucauuuggGcuuuucu.........................................................   1          1           seq
    ...................................aaguucauuuggGcuuuucugu.......................................................   11         1           seq
    ......................................................................agaaucugaaugaacuuuuugg....................   2          0           seq
    ..........................................................................Ccugaaugaacuuuuuggccaau...............   1          1           seq
```
